# Nanoarchitectonics of Spherical Nucleic Acids with Biodegradable Polymer Cores: Synthesis and Evaluation

**DOI:** 10.3390/ma15248917

**Published:** 2022-12-13

**Authors:** Radostina Kalinova, Kirilka Mladenova, Svetla Petrova, Jordan Doumanov, Ivaylo Dimitrov

**Affiliations:** 1 Institute of Polymers, Bulgarian Academy of Sciences, Akad. G. Bonchev St., bl. 103-A, 1113 Sofia, Bulgaria; 2 Faculty of Biology, Sofia University “St. Kliment Ohridski”, 8 Dragan Tzankov Blvd., 1164 Sofia, Bulgaria

**Keywords:** spherical nucleic acids, polyesters, multifunctional, biodegradable, “click” chemistry, oligonucleotides, cytotoxicity

## Abstract

Spherical nucleic acids (SNAs) have gained significant attention due to their unique properties allowing them to overcome the challenges that face current nanocarriers used for gene therapies. The aim of this study is to synthesize and characterize polymer–oligonucleotide conjugates of different architecture and to evaluate the possibility of forming SNAs with biodegradable cores. Initially, two types of azide (multi)functional polyester-based (co)polymers were successfully synthesized and characterized. In the next step, short oligonucleotide strands were attached to the polymer chains applying the highly efficient and metal-free “click” reaction, thus forming conjugates with block or graft architecture. Both conjugates spontaneously self-assembled in aqueous media forming nanosized SNAs with a biodegradable polyester core and a surface of oligonucleotide chains as evidenced from dynamic and electrophoretic light scattering measurements. The nano-assemblies were in vitro evaluated for potential cytotoxicity. Furthermore, the interactions of the newly synthesized SNAs with membrane lipids were studied. The preliminary results indicate that both types of polymer-based SNAs are good candidates for potential application in gene therapy and that it is worth to be further evaluated.

## 1. Introduction

Spherical nucleic acids (SNAs) are an attractive new class of nanomaterials that have been widely investigated in recent decades in terms of treating a variety of diseases via gene regulation [1,2,3,4]. They represent three-dimensional nanostructures consisting of nanoparticle core functionalized with a dense and radially oriented layer of duplexed or single-stranded oligonucleotides [5]. The spherical structure imparts a set of unique properties that are distinct from the properties of conventional linear nucleic acids, such as enhanced nucleotide stability and cellular uptake, improved binding affinity, ability to overcome different biological barriers (epidermal, blood–brain, and blood–tumor barriers), lowered immune responses [1,6,7]. The SNAs are able to actively diffuse through the extracellular matrix and traverse cell membranes without the use of transfection agents.

The first reported SNAs were thiolated oligonucleotides adsorbed onto the surface of gold nanoparticles (AuNP) [5]. Since then, a wide variety of inorganic and organic materials have been used to serve as a core, including silver [8,9], iron oxide [10], platinum [11], silica [12], quantum dots [13], liposomes [14,15,16,17], polymers [18,19,20,21,22], and proteins [23]. The unique chemical and physical properties of SNA in biological environments originate from the densely organized oligonucleotides on the nanoparticle surface, but the right choice of nanoparticles core plays an important role in the design of the nanosystems, as the core determines the sizes and shapes, as well as plasmonic, catalytic, and optical properties of the SNA. Metal nanoparticles have been widely used as SNA core in the last decades, but concerns about their potential long-term toxicity and metabolic fate have encouraged the development of nanostructures with biocompatible and biodegradable organic core, such as liposomes, proteins, and polymers. The liposomal and protein functionalized oligonucleotides are very promising candidates in gene regulation, immunotherapy and intracellular detection. An alternative approach is the use of polymer–oligonucleotide conjugates (POC). The polymer chemistry with its almost limitless capacity for utilizing versatile monomers offers a possibility for the development of synthetic polymers with enriched chemical structure, tunable properties, and even greater tailorability. The coupling reactions, such as Michael addition [24], copper-catalyzed [25] or copper-free cycloaddition [26], amidation [27], and disulfide bond formation [28], are the most direct methods to synthesize POC. However, only a limited number of investigations have been focused on the preparation of SNA with dense layer of nucleic acids attached to the polymer core surface. SNA with the self-immolative poly(carbamate) core covered with a dense DNA shell have been reported by Fukumoto et al. [29]. Polyesters such as poly(ε-caprolactone) (PCL) and poly(lactic-*co*-glycolic acid) (PLGA) have been used to prepare SNAs as well. Zhang et al. synthesized DNA-brush block copolymer micelle (DBBC) via a copper-free strain-promoted azide-alkyne cycloaddition between azide-terminated PCL and dibenzocyclooctyne (DBCO) functionalized DNA [19]. They have managed to prepare SNAs with an increased surface density, a more negatively charged surface and more effective transfection agent-free cellular uptake. The SNAs with biodegradable PLGA-core have been obtained and loaded with the hydrophobic model drug, coumarin 6 [20]. The drug-release kinetics of the prepared nanoconstruct can be independently tuned which may be useful for developing combination therapeutics. Nucleic acid–polymer conjugates synthesized via “click” coupling reactions between poly(ethoxyethyl glycidyl ether) with clickable alkyne end group and azido functionalized hydrophilic oligonucleotide strand, have been recently reported [22]. The corresponding spherical nucleic acids with densely arranged oligonucleotide strands at the surface are non-toxic, with enhanced cellular uptake and nuclease stability.

Biocompatibility and biodegradability are the main requirements for clinical application of SNAs. Therefore, the aim of the current work is to prepare novel SNA nanostructures comprised of polyester and polyester-polycarbonate nanoparticle cores. Poly(lactic acid) (PLA) is one of the most used FDA-approved biopolymers for the preparation of vaccine, drug and gene delivery systems [30]. On the other hand, during the past several years, polycarbonates (PCs) exhibiting excellent biocompatibility, nontoxic degradation products, and tunable mechanical properties have emerged as the next generation drug and gene delivery material [31]. Thus, we present a synthetic strategy for the preparation of SNAs from azide end-functionalized PLA homopolymer or azide multifunctionalized poly(d,l-lactic acid)-*co*-poly(2,2-bis(bromomethyl) trimethylene carbonate) (PLA-*co*-PBMTC) copolymer and DBCO-terminated oligonucleotides via copper-free cycloaddition. The spontaneous self-assembly of both hybrid conjugates in aqueous media is investigated. The average diameters and surface charge of the obtained SNAs are estimated via dynamic light scattering, whereas their morphology is visualized via transmission electron and atomic force microscopies. The initial in vitro evaluations such as cytotoxicity and interaction with membrane lipids are also performed.

## 2. Materials and Methods

### 2.1. Materials and Reagents

All chemicals were purchased from Sigma-Aldrich (St. Louis, MO, USA) unless otherwise stated. Tetrahydrofuran (THF, >99%) and *N,N*-dimethylformamide (DMF, ≥99.5%) were distilled from calcium hydride prior to use. Toluene (ACS reagent, ≥99.5%) was distilled from sodium and benzophenone. d,l-lactide (LA) was recrystallized from toluene/ethyl acetate mixture (95:5 *v*/*v*). Triethylamine (TEA, 99%) was distilled from potassium hydroxide. 2,2-Bis(bromomethyl)-1,3-propandiol (98%), ethyl chloroformate (97%), 2-bromoethanol (95%), tin(II) 2-ethylhexanoate (Sn(Oct)_2_, 92.5–100.0%), sodium azide (≥99.5%), diethyl ether (Et_2_O, for analysis), dichloromethane (DCM, ≥99.5%), methanol (≥99.6%), and dimethyl sulfoxide (DMSO, for analysis EMSURE^®^ ACS) were used as received. The oligonucleotides with the following composition (5′→3′): DBCO-(EG)_4_-(spacer 18)_1_ ta ata cga ctc act ata ggg (DBCO-PEG-oligo) and molar mass *M*_w_ = 7170 g mol^−1^ determined by MALDI-TOF mass spectrometry (Figure S1) were purchased from Biomers.net GmbH (Ulm, Germany).

2,2-Bis(bromomethyl) trimethylene carbonate (BMTC) was synthesized according to already described procedure [32]. Briefly, 2,2-bis(bromomethyl)-1,3-propandiol (3 g, 11.5 mmol) and ethyl chloroformate (3.1 g, 18.8 mmol) were dissolved in 70 mL of dry THF. The solution was cooled down to 0 °C and triethylamine (4.8 mL, 34.5 mmol) was added dropwise. The reaction was allowed to proceed at room temperature for 10 h. Then, the solvent was evaporated and the crude product was purified by recrystallization from THF and diethyl ether (1:4 by volume). Yield: 40%. ^1^H NMR (600 MHz, CDCl_3_, δ, ppm): 4.40 (s, C*H*_2_O, 4H), 3.57 (s, C*H*_2_Br, 4H).

### 2.2. Synthesis of Azido-Terminated Poly(d,l-Lactide) (PLA-N_3_)

#### 2.2.1. Synthesis of Bromo-Terminated Derivative (PLA-Br)

d,l-lactide (1.85 g, 12.8 mmol) was dried in vacuo for 1 h. Then, 5 mL of dry toluene were added under Ar atmosphere. The temperature was adjusted to 90 °C and 0.59 g (0.15 mmol) Sn(Oct)_2_ dissolved in dry toluene (1 mL) were injected. Finally, 2-bromoethanol (0.083 g, 0.37 mmol) was added dropwise. The reaction proceeded for 24 h under argon atmosphere. After polymerization completion, the solvent was evaporated, the product was redissolved in DCM and precipitated in cold methanol. Yield: 1.3 g (70%). ^1^H NMR (600 MHz, CDCl_3_, δ, ppm): 5.25–5.15 (m, C*H*–(CH_3_)–O), 4.51–4.47 (m, C*H*–(CH_3_)–OH), 4.44–4.35 (m, C*H*_2_–O), 3.50 (t, C*H*_2_Br), 1.62–1.54 (m, CH–(C*H*_3_)), 1.49–1.47 (m, CH–(C*H*_3_)–OH).

#### 2.2.2. Nucleophilic Substitution of Alkyl Bromide Polymer Terminal Group with Azide

PLA-Br (0.55 g, 0.11 mmol) and NaN_3_ (0.109 g, 1.7 mmol) were dried in vacuo for 1 h. Then, 6 mL of dry DMF was added under Ar atmosphere. The mixture was stirred at 70 °C for 24 h. After the reaction completion, most of the solvent was removed under a reduced pressure. The product was redissolved in dichloromethane and precipitated in cold methanol. Yield: 0.22 g (40%). ^1^H NMR (600 MHz, CDCl_3_, δ, ppm): 5.24–5.13 (m, C*H*–(CH_3_)–O), 4.40–4.18 (m, C*H*–(CH_3_)–OH + C*H*_2_–O), 3.55–3.46 (m, C*H*_2_N_3_), 1.61–1.53 (m, CH–(C*H*_3_)), 1.48–1.43 (m, CH–(C*H*_3_)–OH). FTIR (1/λ, cm^−1^): 2108 (ν azide), 1747 (ν C=O), 1452 (δ_as_ CH_3_), 1184 (ν_as_ C–O–C), 1082 (ν_s_ C–O–C).

### 2.3. Synthesis of Azido-Terminated Poly(d,l-Lactic Acid)-co-Poly(2,2-Bis(azidomethyl) Trimethylene Carbonate) (N_3_-PLA-co-PAMTC)

#### 2.3.1. Synthesis of Poly(d,l-Lactic Acid)-*co*-Poly(2,2-Bis(bromomethyl) Trimethylene Carbonate) (PLA-*co*-PBMTC)

d,l-lactide (1.0 g, 6.9 mmol) and BMTC (0.28 g, 0.99 mmol) were dried in vacuo for 1 h. Then, 2 mL of dry toluene were added under Ar atmosphere. The temperature was adjusted to 90 °C and after the monomers’ complete dissolution, 0.04 g (0.1 mmol) of Sn(Oct)_2_ dissolved in dry toluene (1 mL) was injected. Finally, 2-bromoethanol (0.03 g, 0.25 mmol) was added dropwise. The reaction was completed in 24 h under argon atmosphere. The toluene was evaporated, the crude product was redissolved in DCM and precipitated in cold methanol. Yield: 80%. ^1^H NMR (600 MHz, CDCl_3_, δ, ppm): 5.24–5.13 (m, C*H*–(CH_3_)–O, PLA), (4.50–4.33 (m, C*H*_2_–O) + (m, C*H*–(CH_3_)–OH), PLA), 4.32–4.18 (O–C*H*_2_, PBMTC), 3.54–3.47 (C*H*_2_Br, PLA + C*H*_2_Br, PBMTC), 1.61–1.52 (m, C*H*–(CH_3_), PLA), 1.50–1.45 (m, CH–(C*H*_3_)–OH, PLA).

#### 2.3.2. Multi Azide-Functionalization of PLA-*co*-PBMTC Copolymer

PLA-*co*-PBMTC (0.3 g, 0.13 mmol –CH_2_Br) and NaN_3_ (0.174 g, 2.7 mmol) were dried in vacuo for 1 h followed by the addition of dry DMF (6 mL) under Ar atmosphere. The mixture was stirred for 24 h at 50 °C. The solvent was removed under reduced pressure. The product was dissolved in dichloromethane and isolated after precipitation in cold methanol. Yield: 33%. ^1^H NMR (600 MHz, CDCl_3_, δ, ppm): 5.25–5.10 (m, C*H*–(CH_3_)–O, PLA), (4.42–4.32 (m, C*H*_2_–O) + (m, C*H*–(CH_3_)–OH), PLA), 4.30–4.05 (O–C*H*_2_, PAMTC), 3.58–3.40 (C*H*_2_N_3_, PLA + C*H*_2_N_3_, PAMTC), 1.60–1.50 (m, C*H*–(CH_3_), PLA), 1.48–1.42 (m, CH–(C*H*_3_)–OH, PLA). FTIR (1/λ, cm^−1^): 2110 (ν azide), 1747 (ν C=O), 1452 (δ_as_ CH_3_), 1184 (ν_as_ C–O–C), 1082 (ν_s_ C–O–C).

### 2.4. Oligonucleotide Conjugation

An identical for both azide containing (co)polymers procedure was applied. Initially, the azide functionalized (co)polymers were dissolved in a mixture of DMSO and DMF (1:1, *v*/*v*, 0.15 mM). Then, a two-fold molar excess from DBCO-PEG-oligo solution in DMSO (0.5 mM) with respect to the azide groups in PLA-N_3_ and N_3_-PLA-*co*-PAMTC, respectively, was added to the polymers’ solutions. The reactions were allowed to proceed for 48 h under argon atmosphere at 40 °C. The obtained polymer–oligonucleotide conjugate solutions were further used for micelles-SNAs formation.

### 2.5. Preparation of SNA-Micelles

The obtained polymer–oligonucleotide conjugates in organic solvent mixtures were dialyzed against water for 4 days at room temperature using dialysis membranes with molecular weight cut-off (MWCO) 50,000 Da. The obtained aqueous dispersions of PLA-SNA and PLA-*co*-PC-SNA were passed through a 0.22 µm syringe filters prior to further analyses.

### 2.6. Characterization Methods

^1^H NMR spectra were recorded on a Bruker Avance II+ spectrometer (Bruker, Billerica, MA, USA) at 600 MHz using CDCl_3_ as a solvent. UV/Vis spectra were taken on a DU 800 Beckman Coulter spectrometer (Beckman Coulter, Inc., Brea, CA, USA). The gel permeation chromatography (GPC) was carried out in tetrahydrofuran at a flow rate of 1.0 mL min^−1^ with Shimadzu Nexera XR HPLC chromatograph (Shimadzu, Kyoto, Japan), equipped with quaternary pump, degasser, automatic injector, column heater, UV/Vis (SPD-20A) detector, differential refractive index (RID-20A) detector, 10 µm PL gel mixed-B, 5 µm PL gel 500 Å and 50 Å columns. The system was calibrated versus polystyrene narrow molar mass standards. Transmission electron microscope (TEM) HRTEM JEOL JEM-2100 (200 kV) instrument (JEOL, Peabody, MA, USA) equipped with CCD camera GATAN Orius 832 SC1000 (Pleasanton, CA, USA) and GATAN Microscopy Suite Software was used to observe morphology and particle size of the SNA micelles. Atomic force microscope (AFM) images were taken on a Bruker NanoScope V9 instrument (Bruker, Billerica, MA, USA) with a 1.00 Hz scan rate under ambient conditions. Observations were performed in ScanAsyst (Peak Force Tapping) mode (Bruker, Billerica, MA, USA). The average diameters and particle size distribution of the prepared SNAs were determined by dynamic light scattering (DLS) using a NanoBrook Plus PALS instrument (Brookhaven Instruments, New York, NY, USA), equipped with a 35-mW solid-state laser operating at λ = 660 nm at a scattering angle of 90°. The average hydrodynamic diameters (*d_H_*) of the hybrid particles were obtained applying the Stokes-Einstein equation:

*d*_*H*_ = *kT*/(3*πηD*), (1)

(*k*—Boltzmann’s constant, *T*—temperature (K), *η*—viscosity, *D*—diffusion coefficient).

The phase analysis light scattering was utilized to determine the electrophoretic mobility of the surface charged SNA-micelles’ dispersions. Thus, the particles’ ζ-potentials were derived by applying the Smoluchowski equation:

*ζ* = 4*πημ*/*ε*, (2)

(*ζ*—zeta potential (mV), *η*—viscosity; *μ*—electrophoretic mobility, *ε*—solvent’s dielectric constant).

The size and size distribution measurements were triplicated per run and were averaged from three independent runs. The zeta potential measurements were also triplicated per run and averaged from twenty runs.

### 2.7. MTT Assay

HepG2 and A549 cells were seeded in 96 wells plate (Corning, Somerville, MA, USA) at a density of 1 × 10^4^ cells per well in DMEM medium with 10% FCS and Penicillin/Streptomycin (Sigma-Aldrich, St. Louis, MO, USA). Cells were treated for 6 h with different concentrations of PLA-SNA and PLA-*co*-PC-SNA (0.1, 0.2, 0.3 and 0.4 µg oligonucleotides in SNAs per 1 × 10^4^ cells). MTT test was performed immediately after the treatment (6 h) and after 24 h and 48 h incubation at 37 °C and 5% CO_2_ in cell medium. The cells were incubated 40 min at 37 °C with MTT solution (3-(4,5-dimethylthiazol-2-yl)-2,5-diphenyltetrazolium bromide, 0.5 g mL^−1^) in DMEM medium without FCS. The MTT formazan product was dissolved in DMSO and the absorbance was detected immediately with Epoch™ Microplate Spectrophotometer (BioTek, Winooski, VT, USA) at λ = 562 nm [33]. Metabolic activity of cells was presented as the ratio: (Absorbance of the treated wells)/(Absorbance of the control wells) × 100%.

### 2.8. Monolayer Experiments

All experiments were performed at identical conditions on a Langmuir trough (Kibron, Inc., Helsinki, Finland) equipped with Wilhelmy dynamometric system. Langmuir films of POPC (1-palmitoyl-2-oleoyl-sn-glycero-3-phosphocholine) at concentrations corresponding to 30 mN m^−1^ surface pressure were used in order to determine the interaction of SNA-micelles with lipid monolayers. POPC was selected and used in these experiments as it is one of the most abundant phospholipids in biological membranes [34,35]. Lipid monolayers were formed by spreading 1 µM POPC on a 150 mM NaCl subphase. Then, micelles’ dispersions with different concentrations (0.1, 0.2, 0.3, or 0.4 µg oligonucleotides in SNAs/500 µL subphase) were added. The surface pressure/concentration (π/C) isotherms were investigated as an indication of the interaction between POPC and SNA-micelles.

## 3. Results and Discussion

### 3.1. Azide-Functional (co)polymers Synthesis and Characterization

**Scheme 1:**
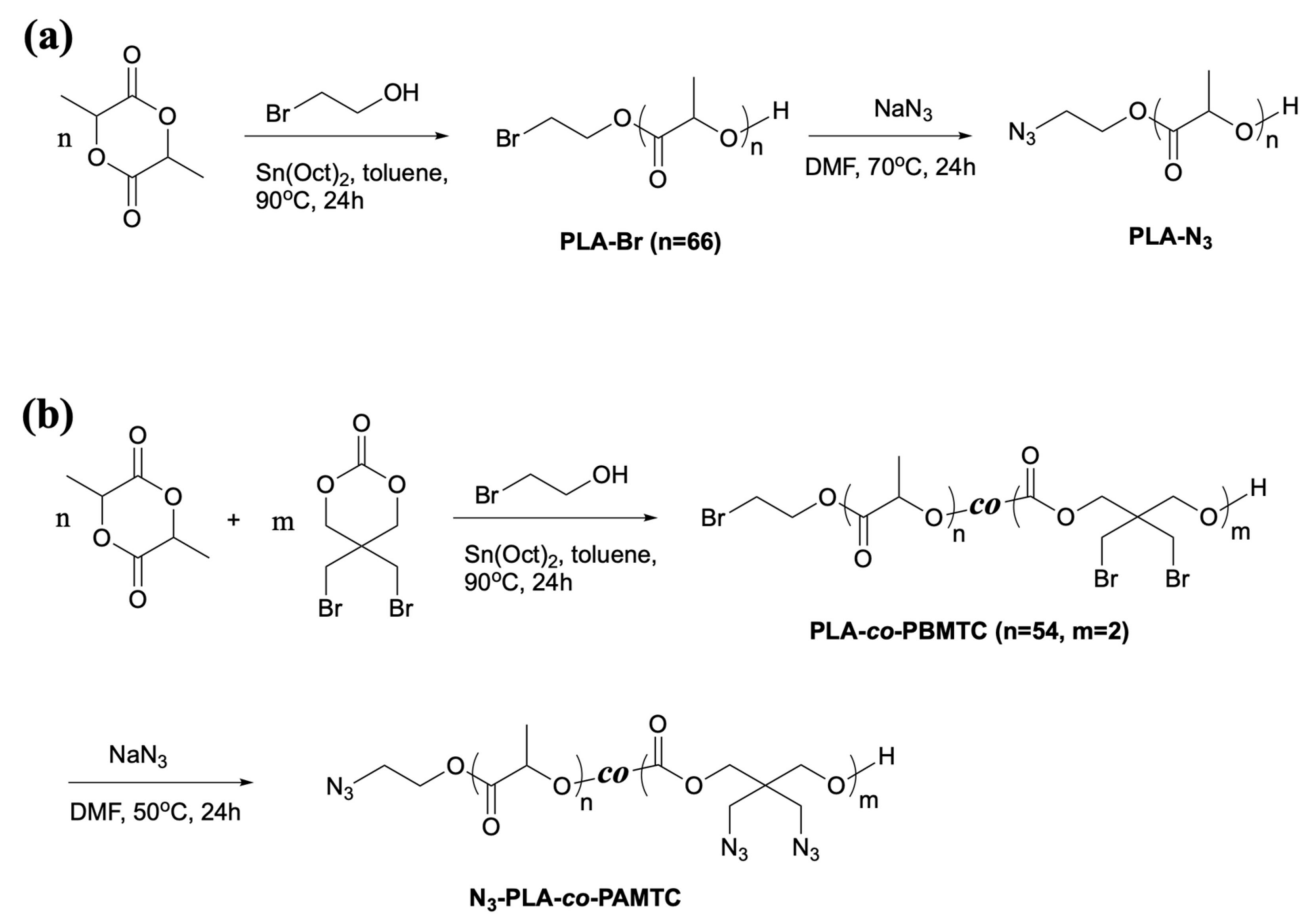
Synthetic routes towards azide-functionalized polyesters: (**a**) azide-terminated polylactide (PLA-N_3_); (**b**) azide-terminated polylactide-*co*-polycarbonate random copolymer modified with azide side groups (N_3_-PLA-*co*-PAMTC).

The novel polymer-based spherical nucleic acids (SNAs) with biodegradable core were prepared applying copper free “click” reactions between specifically synthesized azide functional polylactide-based polymers (PLA-N_3_ or N_3_-PLA-*co*-PAMTC) and oligonucleotides terminated with dibenzocyclooctyne (DBCO) groups. The synthetic strategy towards azide functionalized (co)polymers PLA-N_3_ and N_3_-PLA-*co*-PAMTC is depicted on Scheme 1. Initially, PLA-Br was synthesized by the controlled ring-opening polymerization of d,l-lactide using Sn(Oct)_2_ as the catalyst and 2-bromoethanol as the functional initiator (Scheme 1a). The reaction was performed in toluene at 90 °C. The obtained polyester was characterized by ^1^H NMR spectroscopy. The average degree of LA polymerization was calculated from the relative intensities of methyne protons from the polyester repeating units at 5.16 ppm and those corresponding to the methylene protons next to the bromine end group at 3.50 ppm. The second step involved the preparation of PLA-N_3._ The PLA-Br was reacted with an excess of sodium azide in DMF at 70 °C.

**Figure 1:**
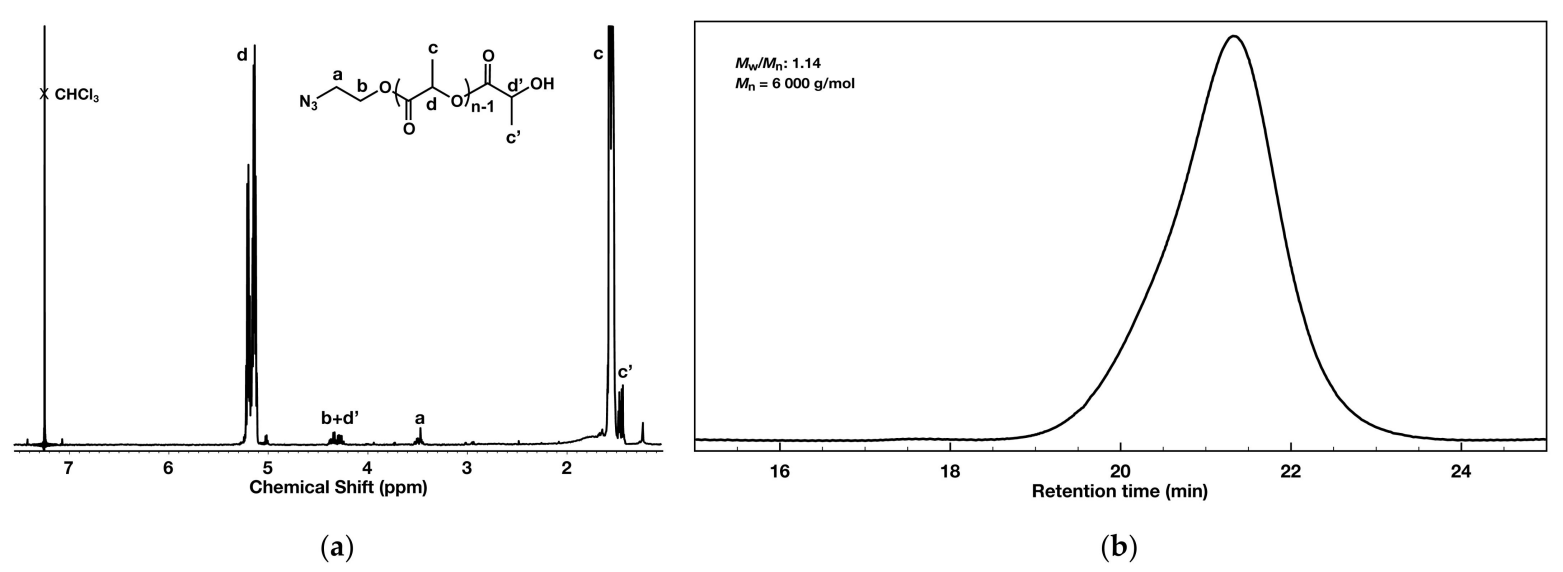
PLA-N_3_ polymer characterization via: (**a**) ^1^H NMR (600 MHz) spectroscopy in CDCl_3_; (**b**) GPC in tetrahydrofuran vs. polystyrene narrow-molar-mass standards.

The nucleophilic substitution of bromine end group by the azide group was evidenced from the product’s ^1^H NMR spectrum. There is a slight upfield shift in the resonance peak corresponding to methylene protons next to the azide end-group at 3.46 ppm as compared to that characteristic for methylene protons next to bromine terminal functionality (Figure 1a).

The successful nucleophilic substitution was further confirmed by the presence of the band at 2110 cm^−1^ corresponding to the azide stretching vibrations in the FTIR-spectrum of the product (not shown). The polymer’s molar-mass distribution was obtained from the GPC-analysis performed in THF. The GPC-elugram revealed the successful preparation of the functional polymer with monomodal molar-mass distribution (*Ð*_M_ = 1.14, Figure 1b).

**Figure 2:**
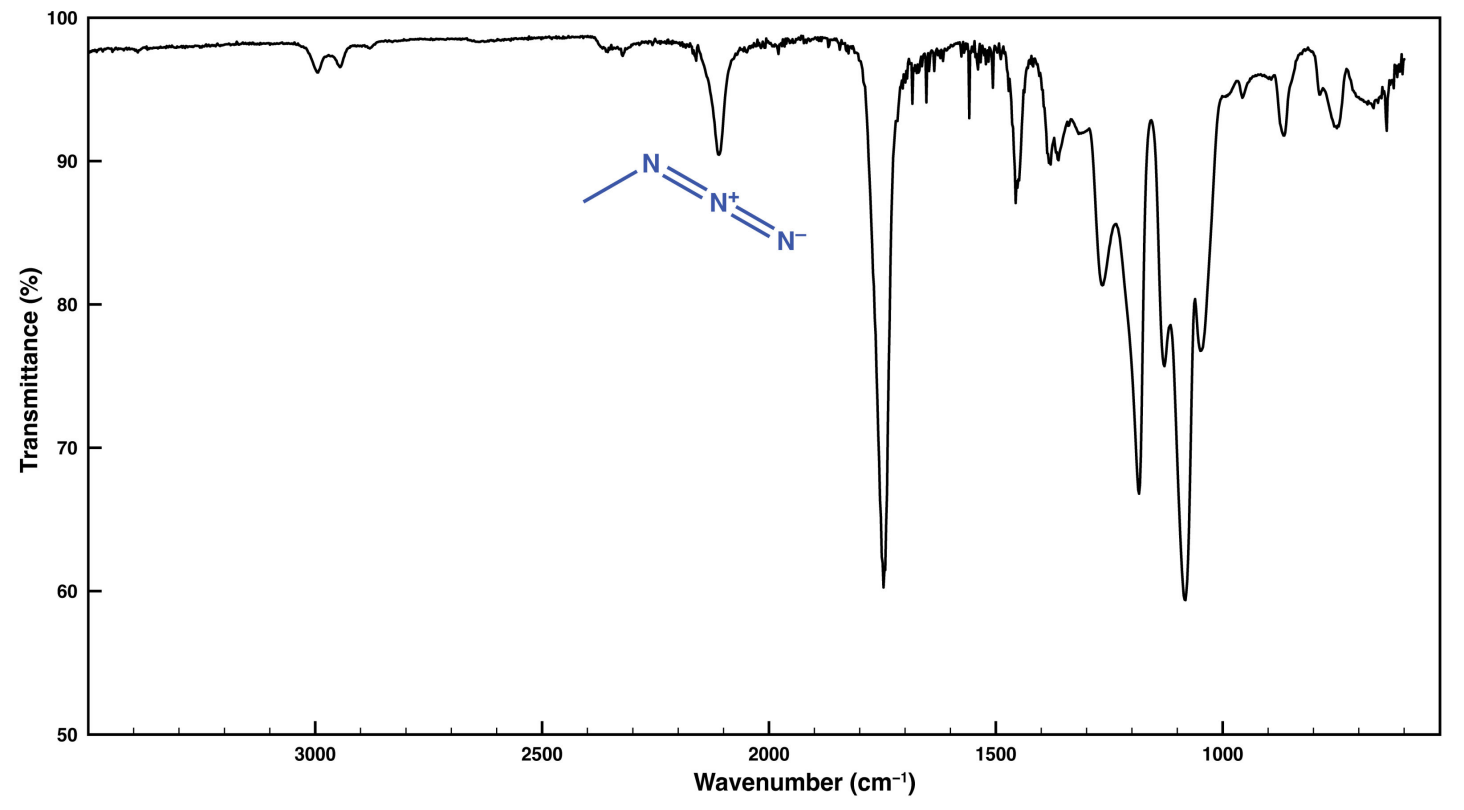
FTIR spectrum of multi-azide-functional N_3_-PLA-*co*-PAMTC copolymer.

The procedure for the synthesis of multifunctional N_3_-PLA-*co*-PAMTC copolymer involved the ring-opening copolymerization of d,l-lactide and the synthesized according to the literature procedure cyclic carbonate BMTC [32]. The composition and purity of BMTC was confirmed by ^1^H NMR spectroscopy (Figure S2). The same functional initiator (2-bromoethanol) and catalyst (Sn(Oct)_2_) were used for the synthesis of multi-bromo-functional biodegradable copolymer (PLA-*co*-PBMTC) (Scheme 1b). The average degree of lactide polymerization was estimated from the ^1^H NMR spectrum of the product from the relative intensities of the methyne protons characteristic for lactide repeating units at 5.20 ppm and the oxymethylene protons of the initiator at 4.50 ppm. The degree of the cyclic carbonate polymerization was estimated from the ratio between the intensities of the two oxymethylene protons from the carbonate repeating units at 4.3 ppm and those of the oxymethylene protons from the initiator at 4.50 ppm. Thus, the copolymer obtained consisted of 54 LA-repeating units with one terminal halide group and two carbonate repeating units with four halide side groups (Scheme 1b). The last synthetic step involved nucleophilic substitution of the halide groups on the copolymer chain by azide groups through reaction with an excess of NaN_3_. The successful substitution reaction was evidenced by FTIR-analysis (Figure 2).

**Figure 3:**
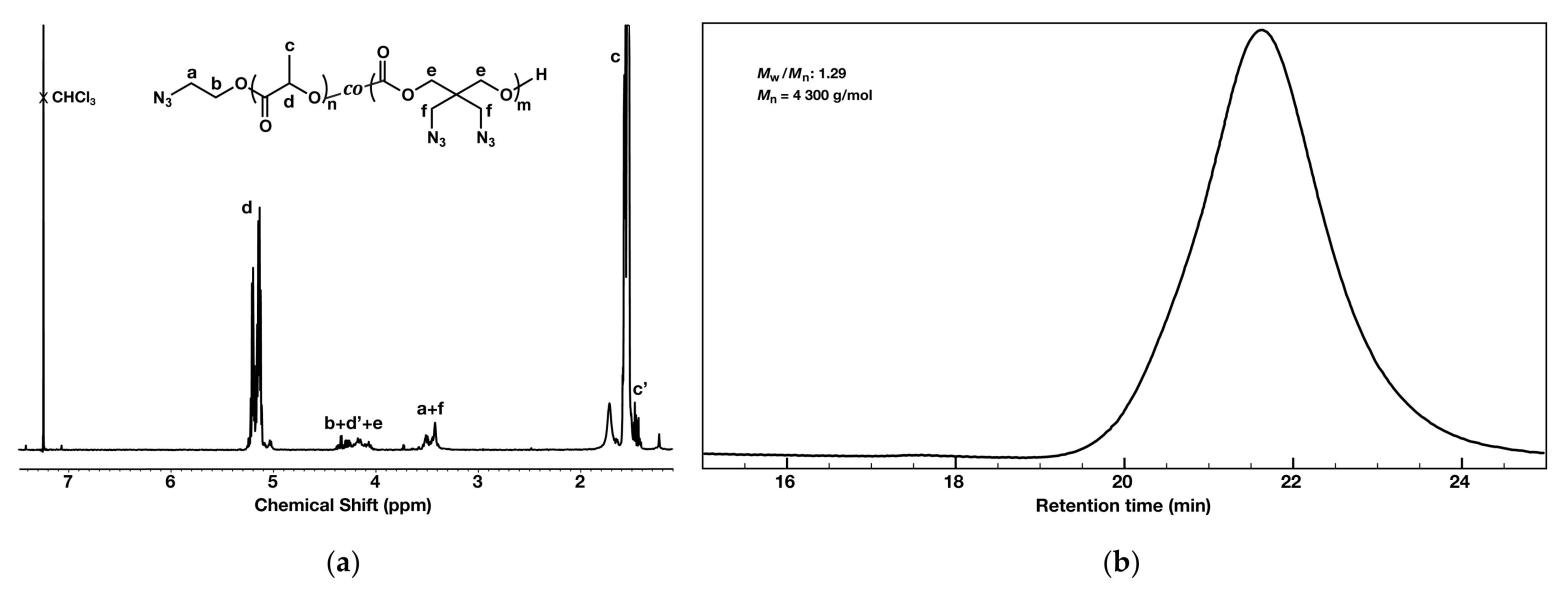
N_3_-PLA-*co*-PAMTC copolymer characterization via: (**a**) ^1^H NMR (600 MHz) spectroscopy in CDCl_3_; (**b**) GPC in tetrahydrofuran vs. polystyrene narrow-molar-mass standards.

The stretching vibration bands at 2110 cm^−1^ characteristic for the azide groups are clearly visible in the spectrum of the final product. Furthermore, the band at 1747 cm^−1^ characteristic for C=O bond stretching of PC and PLA and the band at 1452 cm^−1^ assigned to asymmetric bending vibration of CH_3_ from PLA are also present in the product’s FTIR spectrum. The bands at 1184 and 1082 cm^−1^ correspond to ester C–O–C asymmetric and symmetric stretching vibrations of PLA. The ^1^H NMR spectrum of the copolymer showed an upfield shift for the methylene protons next to the terminal azide (from 3.63 to 3.53 ppm) and the side azide (from 3.50 to 3.43 ppm) groups as a result of the nucleophilic substitution (Figure 3a). GPC analysis revealed the unimodal molar mass distribution curve with polydispersity index of 1.29 (Figure 3b). The estimated number-average molar mass by GPC was in excellent agreement with that calculated from the ^1^H NMR analysis. The molar mass characteristics of the azide-functional (co)polymers obtained are summarized in Table 1.

### 3.2. Oligonucleotide Conjugation and Spherical Nucleic Acids Physico-Chemical Characterization

**Scheme 2:**
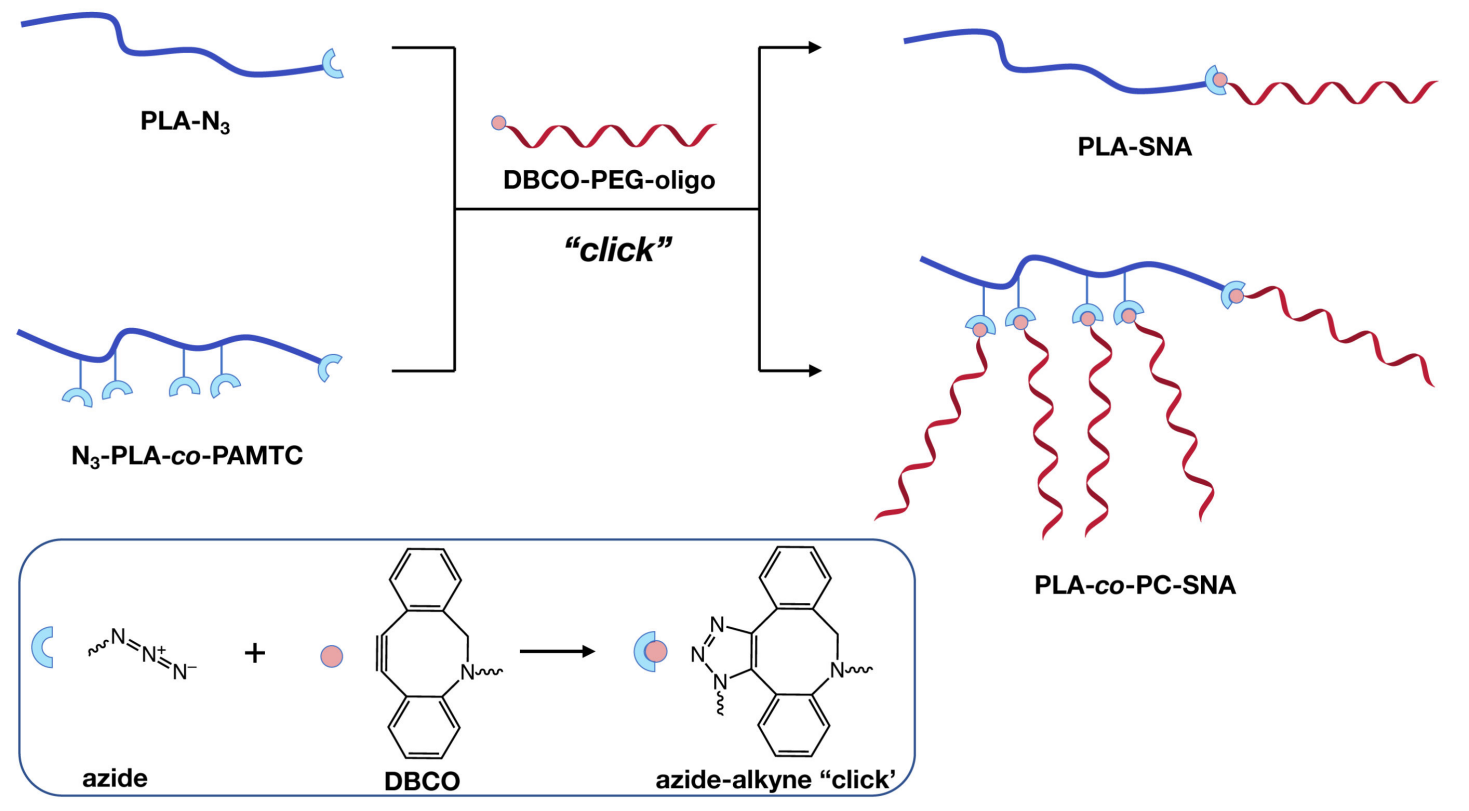
Oligonucleotide-(co)polymer conjugate architectures obtained via copper-free “click” reactions.

The oligonucleotide-(co)polymer conjugates were prepared via highly efficient copper free “click” reaction between the azide functions of N_3_-PLA or N_3_-PLA-*co*-PAMTC and the custom prepared nucleotide sequence with dibenzocyclooctyne terminal group attached through a short PEG-spacer (DBCO-PEG-oligo) according to Scheme 2.

**Figure 4:**
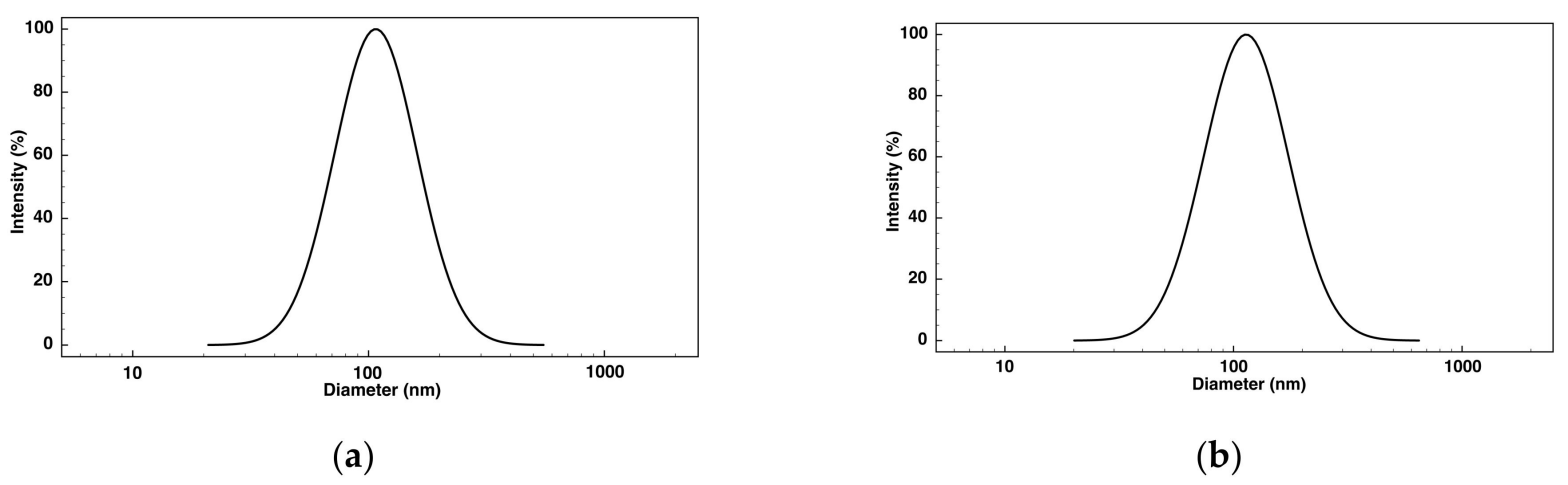
Size distribution curves from DLS measurements of 0.05 mg mL^−1^ aqueous spherical nucleic acid dispersions of: (**a**) PLA-SNA (d = 107 nm, PdI 0.176); (**b**) PLA-*co*-PC-SNA (d = 113 nm, PdI 0.196).

The (co)polymers were dissolved in an organic solvent mixture (DMSO:DMF = 1:1 *v*/*v*) and reacted with the excess of DBCO-PEG-oligo for 48 h at 40 °C. The spherical nucleic acids from both conjugates’ architectures were prepared by the organic solvent mixture gradual replacement with MiliQ^®^ ultra-pure water through dialysis (membrane molecular weight cut-off 50,000 Da). Thus, PLA-SNA and PLA-*co*-PC-SNA spherical nucleic acids dispersed in aqueous media were obtained. The quantitative attachment of DBCO-PEG-oligo to both azido-functional polymers was confirmed from the UV absorbance at λ_max_ = 260 nm (ε = 230,600 M^−1^ cm^−1^) of the respective spherical nucleic acid aqueous dispersions with known concentrations (Figure S3). As a result, knowing the oligonucleotide molar mass, the average molar masses of the (co)polymer–oligonucleotide conjugates were calculated as *M*_n_ = 12,000 g mol^−1^ for the block architecture (PLA-SNA) and *M*_n_ = 40,300 g mol^−1^ for the graft architecture (PLA-*co*-PC-SNA). The two types of spherical nucleic acid dispersions were further characterized by DLS measurements (Figure 4).

The results showed the formation of particles with relatively narrow size distribution for both SNAs with PDI in the 0.176–0.196 range. The average diameters of PLA-SNA and PLA-*co*-PC-SNA micelles were 107 nm and 113 nm, respectively (Table 1). Furthermore, the zeta potential measurements confirmed the presence of negatively charged oligonucleotide strands on the particles’ surfaces. The measured zeta potentials were −12.1 mV for PLA-SNA and −13.9 mV for PLA-*co*-PC-SNA, respectively.

**Figure 5:**
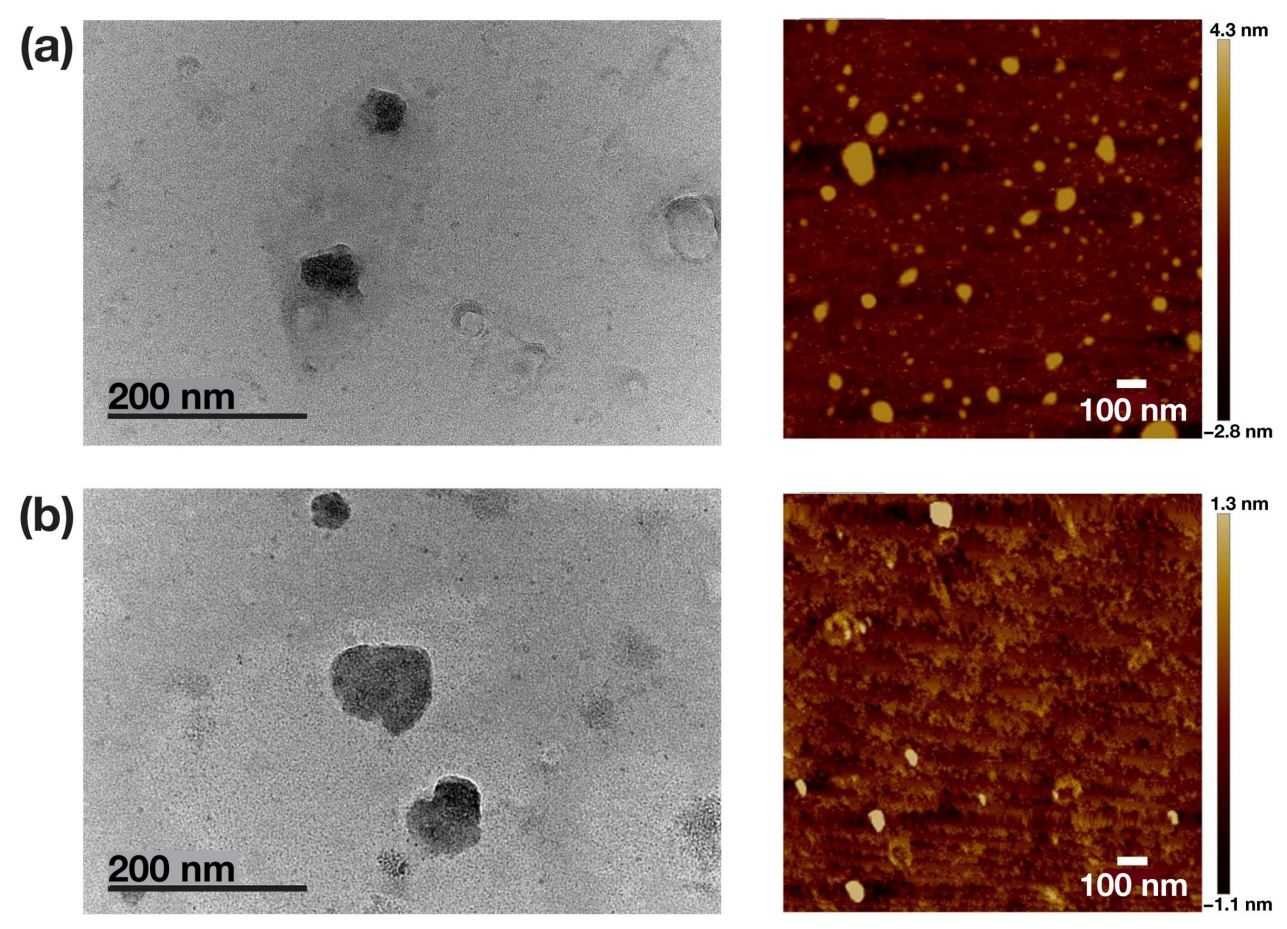
TEM (**left**) and AFM (**right**) images of: (**a**) PLA-SNA; (**b**) PLA-*co*-PC-SNA.

The morphology of the nanoparticles was visualized by transmission electron microscopy (TEM) and atomic force microscopy (AFM) (Figure 5). The images obtained by both methods demonstrated the formation of spherical particles. As expected, the average sizes measured by TEM and AFM on the dry deposits are somewhat smaller than those obtained from the DLS measurements in fully hydrated state. This is most likely due to the particles shrinkage upon drying during the samples’ preparation before the microscopic analyses.

### 3.3. In Vitro Evaluations of PLA-SNA and PLA-co-PC-SNA Spherical Nucleic Acids

**Figure 6:**
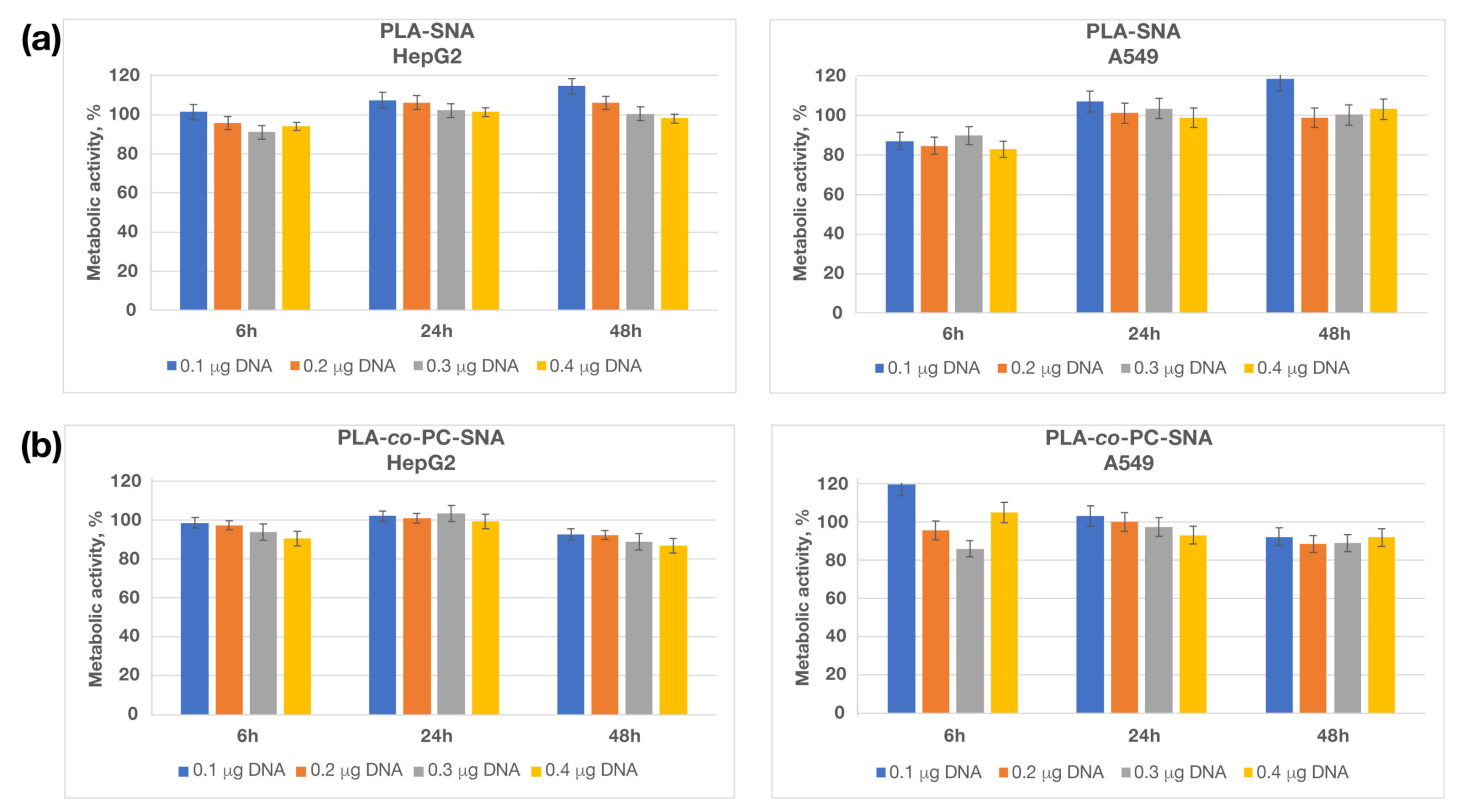
Metabolic activity of HepG2 and A549 cell lines after 6-h incubation with different concentrations of: (**a**) PLA-SNA; (**b**) PLA-*co*-PC-SNA. The MTT assay was performed immediately after treatment with SNAs (6 h), after 24 h and after 48 h. Data are presented as percentage of untreated controls and expressed as a mean ± SE, n = 3.

Cytotoxicity tests are widely used to determine whether the newly synthesized compounds could affect the metabolic activity of cells and if they could cause cellular damage and/or cell death. The results from this study showed that treatment of HepG2 and A549 lines with PLA-SNA and PLA-*co*-PC-SNA had no significant effect on metabolic activity and viability of the cells (Figure 6).

The nanoparticles had minimal cytotoxic potential—the inhibition of metabolic activity was between 2 and 15% versus untreated control in the whole concentration interval studied. Although the differences in the metabolic activity were small, 48 h after treatment PLA-*co*-PC-SNA displayed more evident inhibitory effect in HepG2 and A549 lines than PLA-SNA at the tested concentrations. The data from the MTT assay clearly showed that the nanoparticles did not cause cytotoxic effect, which could make them suitable candidates for future in vivo evaluations and applications. Similar results showing a lack of or minimal cytotoxicity even at high oligonucleotide concentrations and prolonged incubation times were observed by other authors for SNAs comprising various polymeric cores [19,20,36]. The fact that these systems do not need cationic (and often toxic) transfection agents for cell internalization makes them very attractive for potential safe and non-toxic gene regulation.

**Figure 7:**
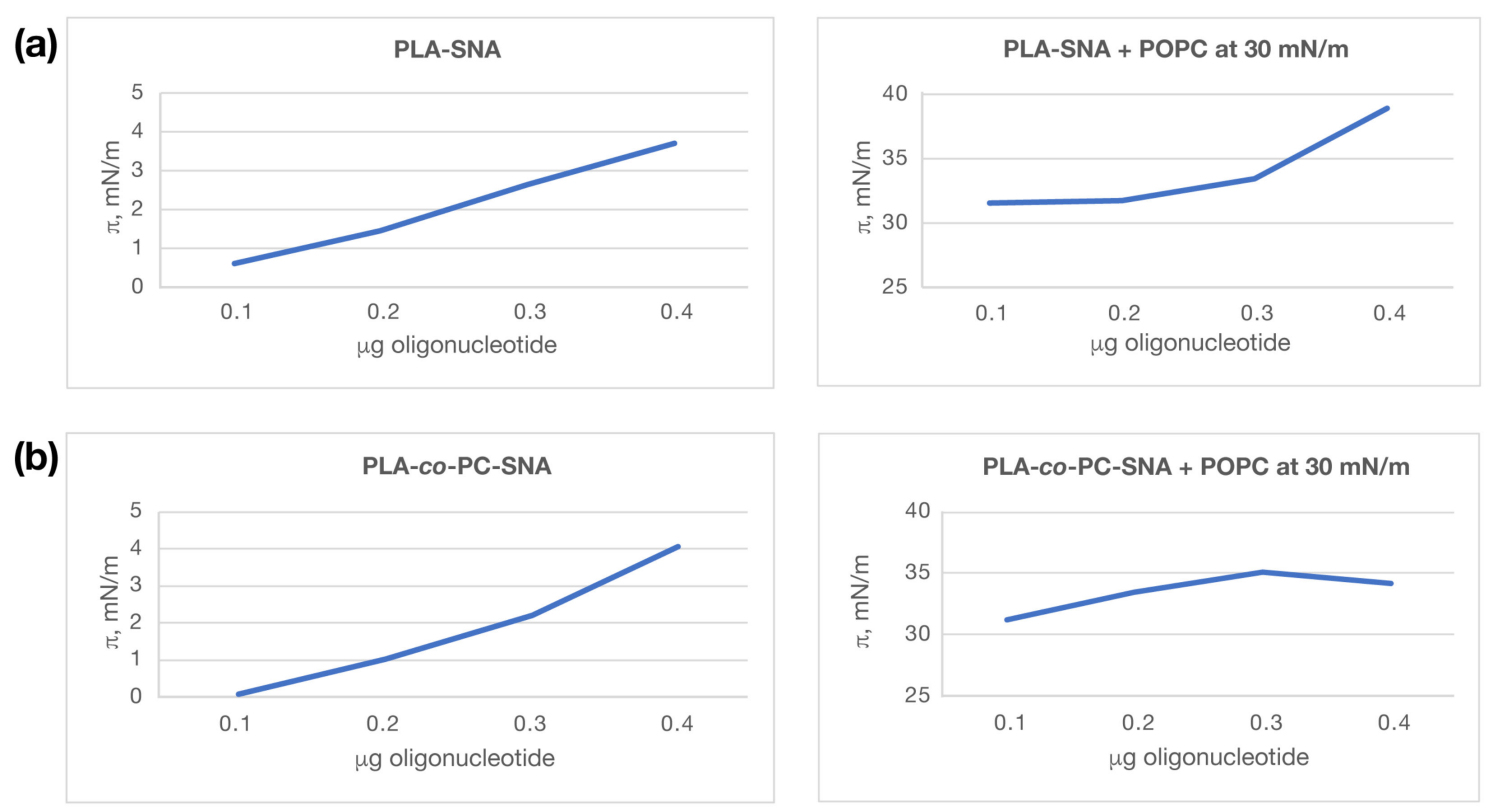
Adsorption π/C isotherms of: (**a**) PLA-SNA and (**b**) PLA-*co*-PC-SNA on 150 mM NaCl without and with POPC.

The fate of SNAs in cells strongly depends on the manner of their internalization across the plasma membrane. An important part of our research was to investigate the interactions of the newly synthesized SNAs with membrane lipids using model membrane systems—Langmuir monolayers. The surface activity of PLA-SNA and PLA-*co*-PC-SNA was evaluated by measuring the adsorption isotherms of their aqueous solutions. The compression/decompression of the formed monolayer was simulated by changing the nanoparticles’ concentration. The adsorption π/C isotherms of PLA-SNA and PLA-*co*-PC-SNA nanoparticles and Langmuir monolayers of SNAs (PLA-SNA and PLA-*co*-PC-SNA) and POPC (with a concentration corresponding to 30 mN/m) are shown in Figure 7. Immediately after the particles were spread, their adsorption began and the surface pressure increased up to about 4 mN/m, which corresponds to their surface activity. The higher the surface activity of the particles, the greater the adsorption on the air/liquid (150 mM NaCl solution) interphase. The relatively low values of surface activity might be due to the poor oligonucleotide compaction, which allows the access of the water molecules to the interior of some SNAs. In our experiments, at the concentrations tested, we did not observe a plateau of π values, which could indicate the critical micelle concentration (CMC). When SNAs were added to POPC films, they induced an increase in π values between 4 and 8 mN/m. The adsorption of nanoparticles to the monolayer depended on their concentration. The electrostatic interactions between the polar head of the phospholipid and the negatively charged SNAs probably results in the formation of surface aggregates and domains of POPC/SNAs, which could explain the changes in π values on the monolayer. Similar interactions of SNAs could also occur with the lipids in the plasma membrane bilayer, which is crucial for their internalization/penetration into cells. Although surface activity makes these nanoparticles promising carrier candidates, their properties need to be further evaluated. Moreover, other types of oligonucleotide-block and graft copolymer conjugates were already used to form SNAs demonstrating enhanced cellular uptake with no need to use cationic transfection reagents [19]. Our preliminary results and those already reported in the literature are optimistic for the potential application of the SNAs with polymer cores in nanomedicine.

## 4. Conclusions

Novel biocompatible and biodegradable SNA nanoparticles were successfully obtained and characterized. The biodegradable (co)polymers bearing azide-end or side functional groups were synthesized via ring-opening (co)polymerization of suitable cyclic monomers. The nucleic acid–polymer conjugates were obtained via copper-free strain-promoted azide-alkyne cycloaddition of DBCO-functionalized oligonucleotides and azide functionalized synthetic polymers. The resulting conjugates spontaneously self-assembled in aqueous media and were prepared by solvent replacement method. The SNAs formed were with negative surface charge and average diameters of around 107 nm for PLA-SNA and 113 nm for PLA-*co*-PC-SNA. Furthermore, the initial cytotoxicity study and interactions of the tested SNA systems with model membrane lipids suggest they are safe and suitable candidates for potential gene regulation and immunotherapy application.

## Figures and Tables

**Table 1:** Characteristics of the azide-functional (co)polymers and the corresponding spherical nucleic acids.

Azide-Functional (co)polymers						Spherical Nucleic Acids		
Sample	DP_LA_^a^	DP_BMTC_^a^	*M*_n_^a^ (g mol^−1^)	*M*_n_^b^ (g mol^−1^)	*Ð*_M_^b^	Sample	d ^c^ (nm)	PdI ^c^
PLA-N_3_	66	-	4800	6000	1.14	PLA-SNA	107	0.176
N_3_-PLA-*co*-PAMTC	54	2	4500	4300	1.29	PLA-*co*-PC-SNA	113	0.196

^a^ Degrees of monomers polymerization (DP_LA_ and DP_BMTC_) and number average molar masses (*M*_n_) determined from the ^1^H NMR analyses in CDCl_3_; ^b^ Molar mass dispersity (*Ð*_M_) and *M*_n_ estimated from the GPC analyses in THF vs. polystyrene standards; ^c^ Average diameters (d) and size distributions (PdI) of spherical nucleic acids determined from the DLS measurements.

## Data Availability

Not applicable.

## Supplementary Materials

The following supporting information can be downloaded at: https://www.mdpi.com/article/10.3390/ma15248917/s1, Figure S1: MALDI-TOF mass spectrum of oligonucleotide with the following composition (5′→3′): DBCO-(EG)_4_-(spacer 18)_1_ ta ata cga ctc act ata ggg (DBCO-PEG-oligo) supplied by Biomers.net GmbH.; Figure S2: ^1^H NMR (600 MHz) spectrum in CDCl_3_ of 2,2-bis(bromomethyl) trimethylene carbonate (BMTC) monomer.; Figure S3: UV/Vis absorption spectra in aqueous media of: (a) poly(d,l-lactide)-based spherical nucleic acids (PLA-SNA); (b) poly(d,l-lactide)-*co*-polycarbonate-based spherical nucleic acids (PLA-*co*-PC-SNA).

## References

[1] Rosi N.L., Giljohann D.A., Thaxton C.S., Lytton-Jean A.K., Han M.S., Mirkin C.A. (2006). Oligonucleotide-modified gold nanoparticles for intracellular gene regulation. Science.

[2] Meckes B., Banga R.J., Nguyen S.T., Mirkin C.A. (2018). Enhancing the stability and immunomodulatory activity of liposomal spherical nucleic acids through lipid-tail DNA modifications. Small.

[3] Zheng D., Giljohann D.A., Chen D.L., Massich M.D., Wang X.-Q., Iordanov H., Mirkin C.A., Paller A.S. (2012). Topical delivery of siRNA-based spherical nucleic acid nanoparticle conjugates for gene regulation. Proc. Natl. Acad. Sci. USA.

[4] Liu H., Kang R.S., Bagnowski K., Yu J.M., Radecki S., Daniel W.L., Anderson B.R., Nallagatla S., Schook A., Agarwal R. (2020). Targeting the IL-17 receptor using liposomal spherical nucleic acids as topical therapy for psoriasis. J. Investig. Dermatol..

[5] Mirkin C.A., Letsinger R.L., Mucic R.C., Storhoff J.J. (1996). A DNA-based method for rationally assembling nanoparticles into macroscopic materials. Nature.

[6] Chinen A.B., Guan C.M., Mirkin C.A. (2015). Spherical nucleic acid nanoparticle conjugates enhance G-quadruplex formation and increase serum protein interactions. Angew. Chem. Int. Ed..

[7] Rouge J.L., Sita T.L., Hao L., Kouri F.M., Briley W.E., Stegh A.H., Mirkin C.A. (2015). Ribozyme–spherical nucleic acids. J. Am. Chem. Soc..

[8] Lee J.-S., Lytton-Jean A.K., Hurst S.J., Mirkin C.A. (2007). Silver nanoparticle-oligonucleotide conjugates based on DNA with triple cyclic disulfide moieties. Nano Lett..

[9] Rische C.H., Goel A., Radovic-Moreno A.F., Gryaznov S.M. (2016). Antibacterial silver core spherical nucleic acids. Mater. Today Commun..

[10] Cutler J.L., Zheng D., Xu X., Giljohann D.A., Mirkin C.A. (2010). Polyvalent oligonucleotide iron oxide nanoparticle “click” conjugates. Nano Lett..

[11] Zhang C., Macfarlane R.J., Young K.L., Choi C.H., Hao L., Auyeung E., Liu G., Zhou X., Mirkin C.A. (2013). A general approach to DNA-programmable atom equivalents. Nat. Mater..

[12] Young K.L., Scott A.W., Hao L., Mirkin S.E., Liu G., Mirkin C.A. (2012). Hollow spherical nucleic acids for intracellular gene regulation based upon biocompatible silica shells. Nano Lett..

[13] Mitchell G.P., Mirkin C.A., Letsinger R.L. (1999). Programmed assembly of DNA functionalized quantum dots. J. Am. Chem. Soc..

[14] Banga R.J., Chernyak N., Narayan S.P., Nguyen S.T., Mirkin C.A. (2014). Liposomal spherical nucleic acids. J. Am. Chem. Soc..

[15] Sprangers A.J., Hao L., Banga R.J., Mirkin C.A. (2017). Liposomal spherical nucleic acids for regulating long noncoding RNAs in the nucleus. Small.

[16] Callmann C.E., Kusmierz C.D., Dittmar J.W., Broger L., Mirkin C.A. (2021). Impact of liposomal spherical nucleic acid structure on immunotherapeutic function. ACS Cent. Sci..

[17] Zhang W., Callmann C.E., Mirkin C.A. (2021). Controlling the biological fate of liposomal spherical nucleic acids using tunable polyethylene glycol shells. ACS Appl. Mater. Interfaces.

[18] Li Z., Zhang Y., Fullhart P., Mirkin C.A. (2004). Reversible and chemically programmable micelle assembly with DNA block-copolymer amphiphiles. Nano Lett..

[19] Zhang C., Hao L., Calabrese C., Zhou Y., Choi C.H., Xing H., Mirkin C.A. (2015). Biodegradable DNA-brush block copolymer spherical nucleic acids enable transfection agent-free intracellular gene regulation. Small.

[20] Zhu S., Xing H., Gordiichuk P., Park J., Mirkin C.A. (2018). PLGA Spherical nucleic acids. Adv. Mater..

[21] Melamed J.R., Kreuzberger N.L., Goyal R., Day E.S. (2018). Spherical nucleic acid architecture can Improve the efficacy of polycation-mediated siRNA delivery. Mol. Ther. Nucleic Acids.

[22] Bakardzhiev P., Toncheva-Moncheva N., Mladenova K., Petrova S., Videv P., Moskova-Doumanova V., Topouzova-Hristova T., Doumanov J., Rangelov S. (2020). Assembly of amphiphilic nucleic acid–polymer conjugates into complex superaggregates: Preparation, properties, and in vitro performance. Eur. Polym. J..

[23] Brodin J.D., Sprangers A.J., McMillan J.R., Mirkin C.A. (2015). DNA-mediated cellular delivery of functional enzymes. J. Am. Chem. Soc..

[24] Isoda K., Kanayama N., Fujita M., Takarada T., Maeda M. (2013). DNA terminal mismatch-induced stabilization of polymer micelles from RAFT-generated poly(*N*-isopropylacrylamide)-DNA block copolymers. Chem. Asian J..

[25] Ni Q., Zhang F., Zhang Y., Zhu G., Wang Z., Teng Z., Wang C., Yung B.C., Niu G., Lu G. (2018). In situ shRNA synthesis on DNA-polylactide nanoparticles to treat multidrug resistant breast cancer. Adv. Mater..

[26] Wang D., Lu X., Jia F., Tan X., Sun X., Cao X., Wai F., Zhang C., Zhang K. (2017). Precision tuning of DNA- and poly(ethylene glycol)-based nanoparticles via coassembly for effective antisense gene regulation. Chem. Mater..

[27] Lee K., Povlich L.K., Kim J. (2007). Label-free and self-signal amplifying molecular DNA sensors based on bioconjugated polyelectrolytes. Adv. Funct. Mater..

[28] Lee S.H., Mok H., Lee Y., Park T.G. (2011). Self-assembled siRNA–PLGA conjugate micelles for gene silencing. J. Control. Release.

[29] Fukimoto S., Kawade M., Kimura K., Akiyama Y., Kikuchi A. (2021). Preparation of spherical nucleic acid nanoparticles containing a self-immolative poly(carbamate) core. Anal. Sci..

[30] Elmowafy E.M., Tiboni M., Soliman M.E. (2019). Biocompatibility, biodegradation and biomedical applications of poly(lactic acid)/poly(lactic-co-glycolic acid) micro and nanoparticles. J. Pharm. Investig..

[31] Chen W., Meng F.H., Cheng R., Deng C., Feijen J., Zhong Z.Y. (2014). Advanced drug and gene delivery systems based on functional biodegradable polycarbonates and copolymers. J. Control. Release.

[32] Zhang X., Zhong Z., Zhuo R. (2011). Preparation of azido polycarbonates and their functionalization via click chemistry. Macromolecules.

[33] Stockert J.C., Horobin R.W., Colombo L.L., Blázquez-Castro A. (2018). Tetrazolium salts and formazan products in Cell Biology: Viability assessment, fluorescence imaging, and labeling perspectives. Acta Histochem..

[34] Mladenova K., Petrova S.D., Georgiev G.A., Moskova-Doumanova V., Lalchev Z., Doumanov J.A. (2014). Interaction of Bestrophin-1 with 1-palmitoyl-2-oleoyl-sn-glycero-3-phosphocholine (POPC) in surface films. Colloids Surf. B.

[35] Andreeva T.D., Petrova S.D., Mladenova K., Moskova-Doumanova V., Topouzova-Hristova T., Petseva Y., Mladenov N., Balashev K., Lalchev Z., Doumanov J.A. (2018). Effects of Ca^2+^, Glu and GABA on hBest1 and composite hBest1/POPC surface films. Colloids Surf. B.

[36] Banga R.J., Meckes B., Narayan S.P., Sprangers A.J., Nguyen S.T., Mirkin C.A. (2017). Cross-linked micellar spherical nucleic acids from thermoresponsive templates. J. Am. Chem. Soc..

