# Supplementary material for: Nanoarchitectonics of Spherical Nucleic Acids with Biodegradable Polymer Cores: Synthesis and Evaluation
Source: Materials (Basel). 2022 Dec 13;15(24):8917. doi: 10.3390/ma15248917 (PMC9786340; doi:10.3390/ma15248917)
Supplement: Supplementary file 1 [file materials-15-08917-s001.zip › materials-2093319-supplementary.pdf]

Supplementary Materials

Nanoarchitectonics of Spherical Nucleic Acids with Biodegradable Polymer Cores: Synthesis and Evaluation

Radostina Kalinova [1,*], Kirilka Mladenova [2], Svetla Petrova [2], Jordan Doumanov [2] and Ivaylo Dimitrov [1,*]

[1] Institute of Polymers, Bulgarian Academy of Sciences, Akad. G. Bonchev St., bl. 103-A, 1113 Sofia, Bulgaria;

[2] Faculty of Biology, Sofia University "St. Kliment Ohridski", 8 Dragan Tzankov Blvd., 1164 Sofia, Bulgaria

*Correspondence: kalinova@polymer.bas.bg; dimitrov@polymer.bas.bg

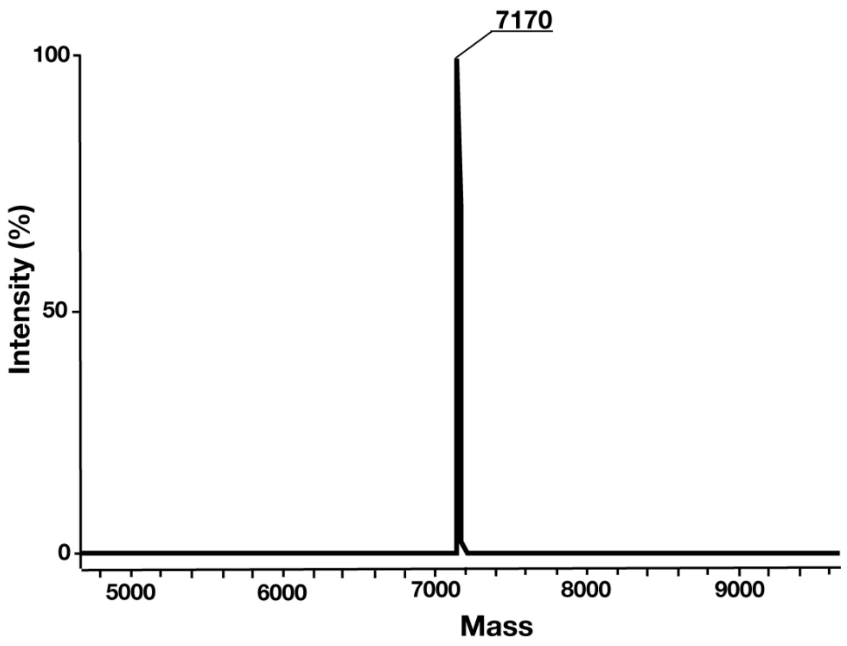

Figure S1. MALDI-TOF mass spectrum of oligonucleotide with the following composition (5'→3'): DBCO-(EG)4-(spacer 18)1 ta ata cga ctc act ata ggg (DBCO-PEG-oligo) supplied by Biomers.net GmbH.

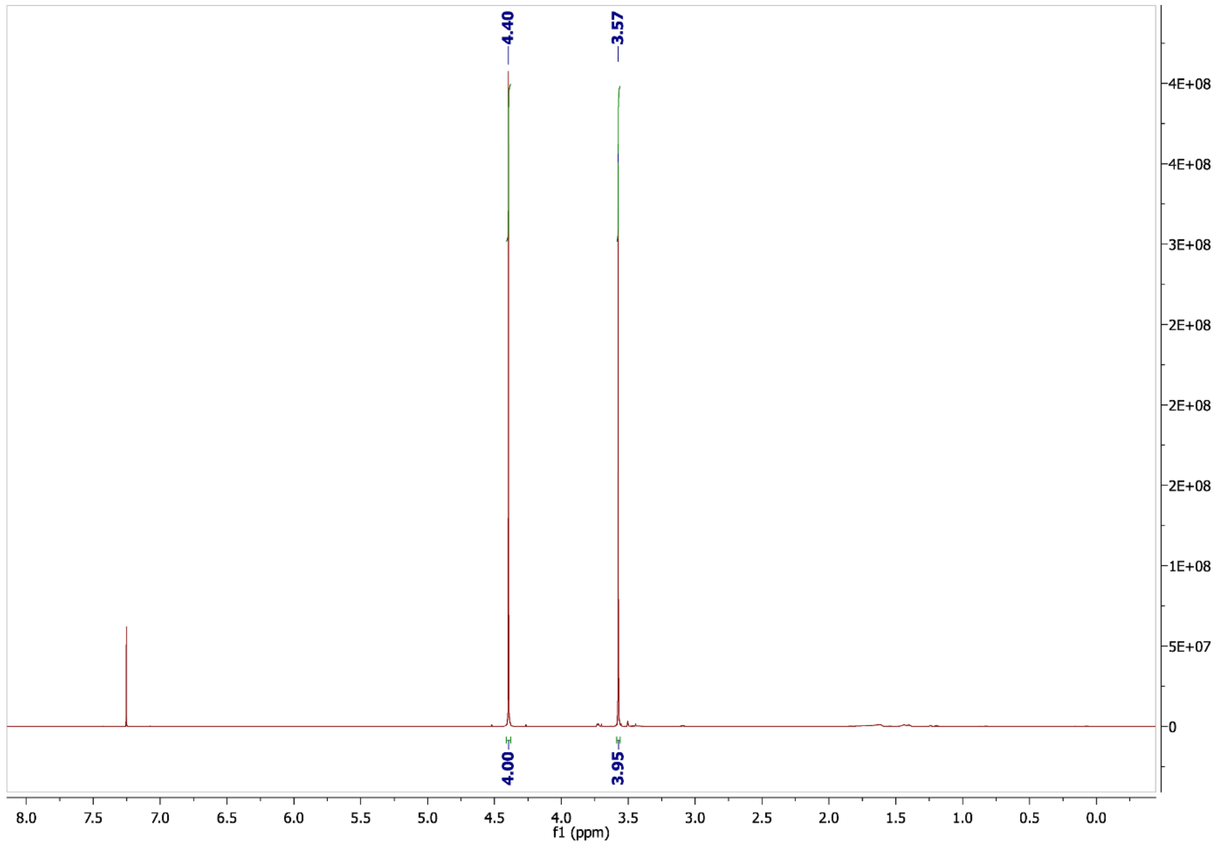

Figure S2. ^{1}H NMR (600 MHz) spectrum in $CDCl_3$ of 2,2-bis(bromomethyl) trimethylene carbonate (BMTC) monomer.

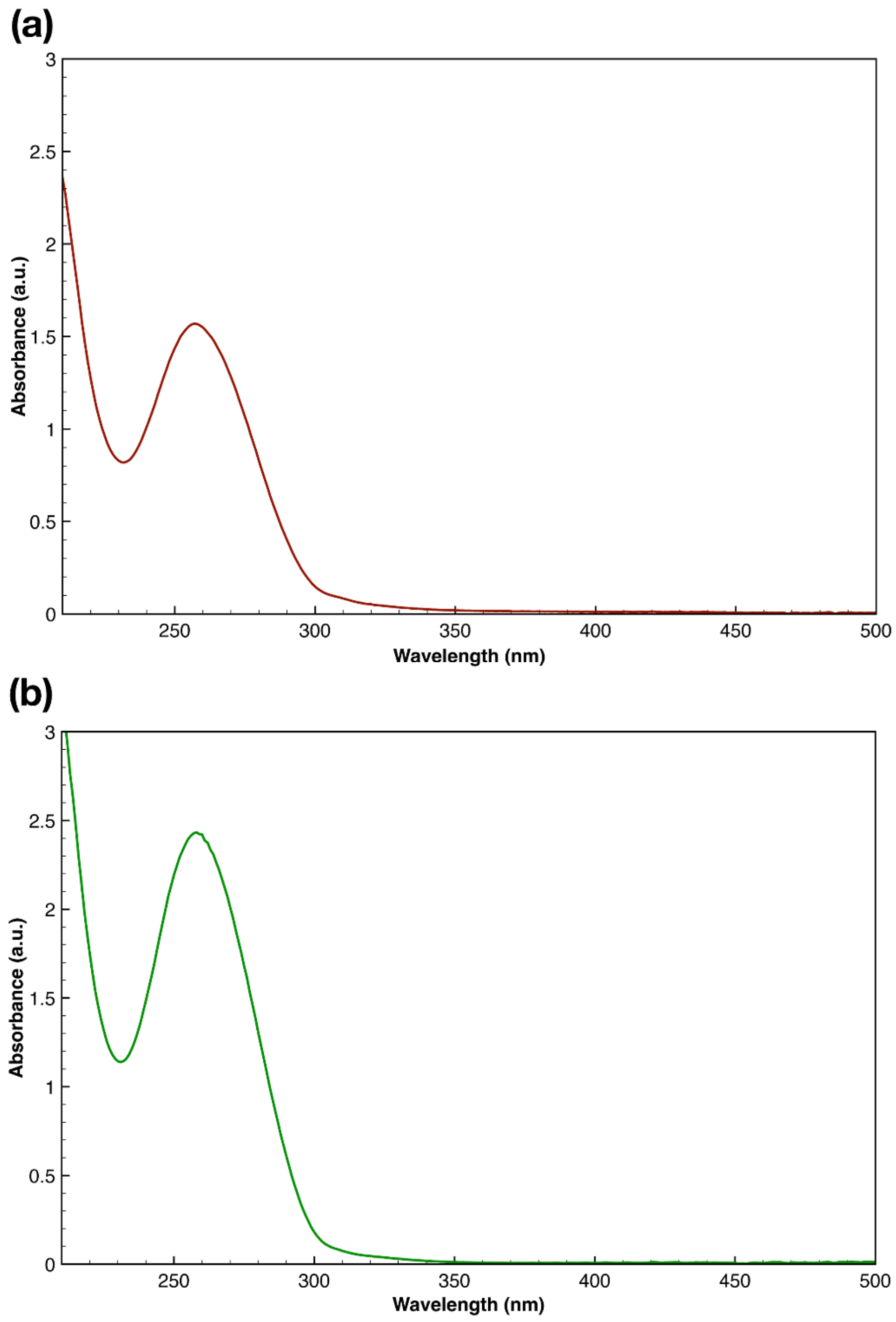

Figure S3. UV/Vis absorption spectra in aqueous media of: (**a**) poly(D,L-lactide)-based spherical nucleic acids (PLA-SNA); (**b**) poly(D,L-lactide)-*co*-polycarbonate-based spherical nucleic acids (PLA-*co*-PC-SNA).
